# O088: Bad design, bad practices, bad bugs – Elizabethkingia meningoseptica outbreak in ICU

**DOI:** 10.1186/2047-2994-2-S1-O88

**Published:** 2013-06-20

**Authors:** S Salmon, M Balm, C Teo, R Mahdi, D Fisher

**Affiliations:** 1 Infection Control Team, National University Hospital Singapore, Singapore, Singapore; 2 Microbiology, National University Hospital Singapore, Singapore, Singapore

## Introduction

*Elizabethkingia meningoseptica* is a nosocomial-adapted, Gram negative bacillus with intrinsic resistance to most antibiotics. An outbreak investigation was commenced when five patients developed *E. meningoseptica* bacteraemia within a three week period in the Cardiothoracic Intensive Care Unit (CTICU) and Surgical ICU (SICU).

## Methods

Analysis of laboratory data, case reviews, workflows within CTICU and extensive environmental sampling of surfaces and taps within four ICU wards, four general wards, two dialysis units and eight operating theatres were performed.

## Results

Upon review laboratory data revealed an unrecognized subtle increasing trend of *E. meningoseptica* clinical infections in all ICUs over the preceding 3 years. *E. meningoseptica* was cultured from aerators of 44% (35/79) taps. Taps in sinks frequently used for non-sanctioned practices were more likely to be contaminated (95% CI 1.2-2.3, p<0.003). *Elizabethkingia* was not cultured from any other surfaces within patient rooms. Investigation of ICU nursing practice revealed introduction of non-sanctioned practices regarding disposal of patient respiratory secretions and cleaning of patient equipment in designated hand hygiene sinks within patient rooms. An urgent education programme was instituted to change these practices. Rooms underwent terminal cleaning. Faucets were systematically cleaned and aerators were changed. No further cases occurred in SICU or CT-ICU over the following three months. One month after aerator change, new aerators remained free of *E. meningoseptica*.

## Conclusion

Introduction of non-sanctioned practices due to suboptimal unit design may have unintended consequences for vulnerable patients. Nursing workflows must also be practical to ensure proper waste disposal and cleaning of medical equipment.

## Disclosure of interest

None declared.

